# Identifying factors that shape whether digital food marketing appeals to children

**DOI:** 10.1017/S1368980023000642

**Published:** 2023-06

**Authors:** Camilo E Valderrama, Dana Lee Olstad, Yun Yun Lee, Joon Lee

**Affiliations:** 1 Department of Applied Computer Science, University of Winnipeg, 515 Portage Avenue, Winnipeg, MB R3B 2E9, Canada; 2 Department of Community Health Sciences, Cumming School of Medicine, University of Calgary, Alberta, Canada; 3 Data Intelligence for Health Lab, Cumming School of Medicine, University of Calgary, Calgary, Alberta, Canada; 4 Department of Cardiac Sciences, Cumming School of Medicine, University of Calgary, Calgary, Alberta, Canada; 5 Department of Preventive Medicine, School of Medicine, Kyung Hee University, Seoul, South Korea

**Keywords:** Food marketing, Digital media, Child appeal, Machine learning

## Abstract

**Objective:**

Children are frequently exposed to unhealthy food marketing on digital media. This marketing contains features that often appeal to children, such as cartoons or bold colours. Additional factors can also shape whether marketing appeals to children. In this study, in order to assess the most important predictors of child appeal in digital food marketing, we used machine learning to examine how marketing techniques and children’s socio-demographic characteristics, weight, height, BMI, frequency of screen use and dietary intake influence whether marketing instances appeal to children.

**Design:**

We conducted a pilot study with thirty-nine children. Children were divided into thirteen groups, in which they evaluated whether food marketing instances appealed to them. Children’s agreement was measured using Fleiss’ kappa and the S score. Text, labels, objects and logos extracted from the ads were combined with children’s variables to build four machine-learning models to identify the most important predictors of child appeal.

**Setting:**

Households in Calgary, Alberta, Canada.

**Participants:**

39 children aged 6–12 years.

**Results:**

Agreement between children was low. The models indicated that the most important predictors of child appeal were the text and logos embedded in the food marketing instances. Other important predictors included children’s consumption of vegetables and soda, sex and weekly hours of television.

**Conclusions:**

Text and logos embedded in the food marketing instances were the most important predictors of child appeal. The low agreement among children shows that the extent to which different marketing strategies appeal to children varies.

Each day, children are exposed to large quantities of marketing for unhealthy food on digital media including websites, social media and online games^(1)^. This high level of marketing exposure shapes children’s dietary preferences for unhealthy foods and brands^(2,3)^. This is concerning as dietary patterns tend to track from childhood to adolescence and through to adulthood, which may increase children’s risk of CVD, diabetes, obesity and poor mental health over the long term^(4–7)^.

To mitigate the long-term consequences of exposing children to unhealthy food marketing, academics, practitioners and public and non-profit organisations have been advocating for regulations to restrict unhealthy food marketing to children^(8)^. However, developing regulations to restrict food marketing to children is challenging because it is unclear what constitutes ‘marketing to children’^(9)^. Various policy documents refer to ‘child targeted’ or ‘child appealing’ food marketing. The former is typically indicated by the use of specific marketing strategies targeting children, such as childish font, cartoons or pictures of children, and is therefore relatively simple to operationalise^(10)^. The latter has a broader definition and can refer to marketing that may not target children directly, but nevertheless attracts their attention and may persuade them to consume a product^(9)^. For instance, Mulligan et al.^(11)^ previously found that children were attracted to health claims on food packaging, a marketing strategy that typically targets adults.

In an attempt to measure the power of food marketing techniques, Elliot and Truman^(12)^, after reviewing eighty studies in the field, found that the use of spokes characters (e.g. cartoons, licenced characters or famous athletes) was a persuasive marketing technique. The authors also reported that the most frequently attractive messages for children were related to health/nutrition^(13)^, taste^(14)^ and fun^(15)^. Spokes characters and messages can be found in the text, labels, objects and logos contained in food marketing instances, thus making food marketing techniques an important determinant of whether marketing appeals to children.

Additional factors can also shape whether marketing appeals to children. For instance, Elliot^(16)^ found that children aged 8–9 years did not like food marketing instances that they perceived to be too childish. Similarly, previous studies have reported that food companies have been using food marketing techniques that may appeal to African American and Hispanic children, such as ethnic symbols, linguistic styles and music^(2,17)^.

Other factors like household income, exposure to screens and nutritional literacy can shape the impact of food marketing on children. Indeed, lower-income children are likely to make more advertising-induced purchases than their higher-income peers^(18)^. Similarly, children’s food choice is affected by exposure to screens^(19)^, such as television^(20)^, advergames^(21)^, internet^(22)^ and social media^(23)^. Children’s nutritional knowledge has also been shown to be inversely related to the consumption of unhealthy foods^(24)^.

However, to date, quantitative studies have not examined the relative importance of marketing features and children’s socio-demographic and other characteristics in shaping which marketing appeals to them. This is challenging to perform using traditional statistical methods, due to limitations associated with handling interactions between multiple predictors simultaneously^(25)^. Machine learning, by contrast, can handle multiple predictors by using mathematical models that extract patterns from data to map such input predictors to a specific outcome^(26,27)^. Hence, this approach opens up opportunities to conduct more comprehensive and complex syntheses of multiple factors that shape the extent to which food marketing appeals to children.

In the current study, in order to assess the most important predictors of child appeal in digital food marketing, we used machine learning to examine how marketing techniques and children’s socio-demographic characteristics, weight, height, BMI, frequency of screen use and dietary intake influence whether marketing instances appeal to children.

## Methods

### Participants and recruitment

We conducted a pilot study with thirty-nine children residing in Calgary, Alberta, Canada, aged 6–12 years, who could speak, read and understand English^(28)^. We recruited the children through social service agencies, online community groups on Facebook (a social media platform) and via an e-mail distributed to primary caregivers with whom the researchers were acquainted. Our goal was to recruit a diverse range of participants with varying socio-economic backgrounds (e.g. household income), ethnicity, body weight status, age, sex and immigrant status.

### Data collection

Before starting a session, caregivers provided written, informed consent for their child to participate, and children also provided assent. Sessions were conducted on a secure online platform (Zoom, with a password required to access the session), guided by an interviewer.

At the beginning of each interview, caregivers and their child answered a questionnaire based on the Canadian Community Health Survey^(29)^ to report the child’s age, sex at birth, ethnicity, immigration status, parental education, height and weight. We also assessed weekly consumption of vegetables, fruits, soda (including regular/diet soda, fruit drinks, 100 % fruit juice, energy drinks, sports drinks) and snack foods (e.g. chips, candy, chocolate, baked goods, desserts)^(30)^, perceived income adequacy^(31,32)^ and screen time (hours spent watching television and using the internet, tablets, and smartphones in the past week) using items from previous surveys^(33,34)^.

Following the questionnaire, caregiver presence was optional for the food marketing labelling session. Caregivers present at the session with their child were asked to refrain from answering for their child. During the session, each child evaluated approximately 104 food marketing ads in two separate sessions of 15 min each (∼52 marketing ads per session). For each marketing instance, children answered a binary question. The question for the first eighteen interviewed children was: ‘Is this ad for kids your age?’. For the remaining twenty-one children, the question was: ‘Is this ad for kids like you?’. The question was changed as some children found it easier to respond to the second question (i.e. they could not determine the intended age demographic for the ads).

### Food marketing ads

**Fig. 1:**
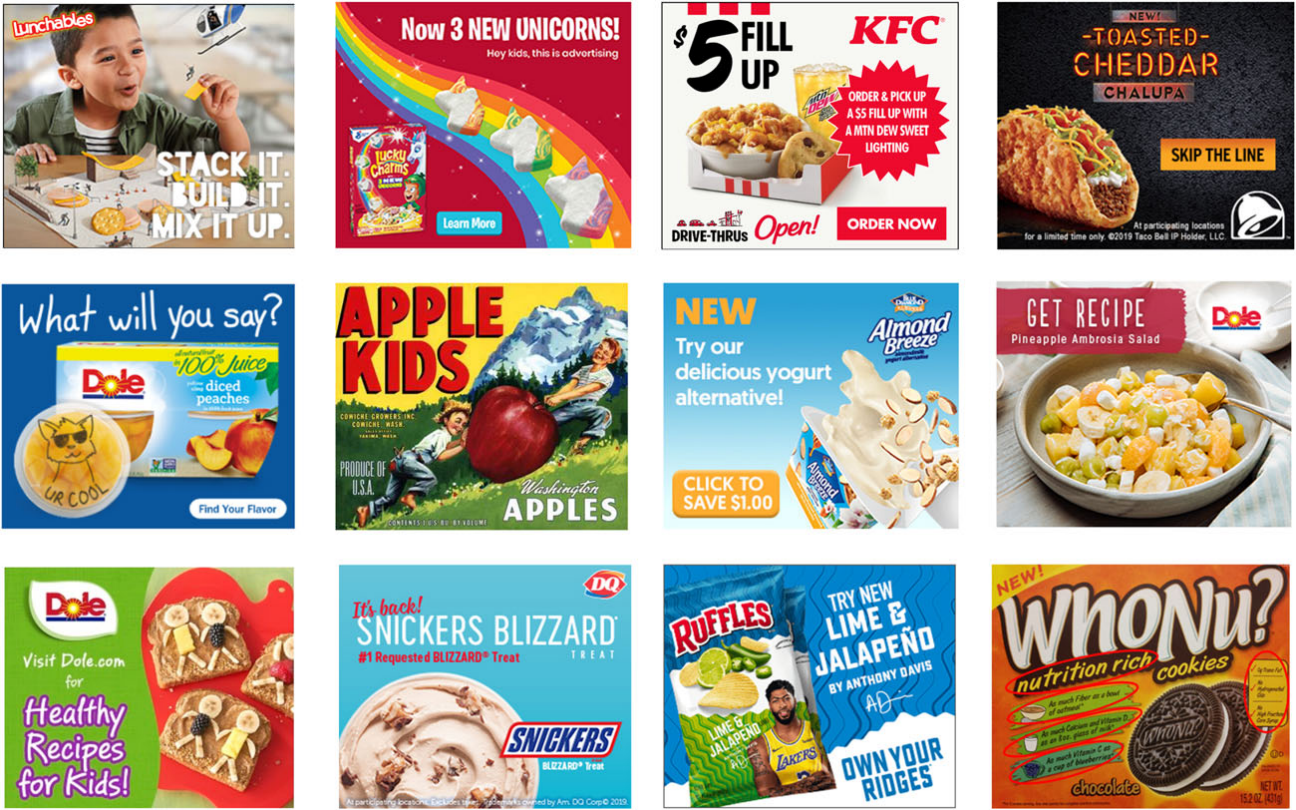
Examples of the food marketing instances used in the study

All the food marketing instances were obtained between September and October 2020 using Oracle Moat^(35)^, a marketing analytics suite in which users can search for ads for specific companies, brands or food categories (e.g. snacks, sodas, breakfast cereal). For each food category listed in Appendix 1, we included ads in English from well-known brands and companies in North America. Figure 1 shows examples of the food marketing instances used in this study, whereas Table 1 shows the ten most common brands featured in the food marketing instances.

**Table 1:** Top ten brands featured in the food marketing instances

Brand	*n*	%
M&Ms	107	7·8
Nature Valley	83	6·1
Kellogg’s	70	5·1
Subway	61	4·5
Wendy’s	55	4·0
McDonald’s	52	3·8
KFC	26	1·9
Frito Lay	26	1·9
Quaker	24	1·8
Pringles	22	1·5

In total 1366 food ads were extracted from Moat. One of these food ads was discarded because it contained an alcoholic product. The remaining 1365 ads were assigned to twenty-six sets, ensuring that each set had a similar proportion of ads containing child-targeting elements (e.g. cartoons, bold colours, fun themes).

The thirty-nine children were randomly divided into thirteen groups of three children each. Each group was assigned to two food ad sets. Each child in the group individually reviewed the two sets of food ads, answering for each ad whether the ad appealed to them (yes or no answer). Therefore, the two sets were labelled by the same three children, and each food ad received three responses (answers). The sets evaluated by the same three children were combined, thus resulting in a total of thirteen shared sets.

### Agreement between children

The sets evaluated by the same three children were combined, resulting in a total of thirteen shared sets. The agreement between children in each shared set was analysed using Fleiss’ kappa statistic^(36)^, and the S score developed by Bennett et al.^(37)^ These two metrics calculate the degree of agreement as:

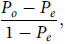

where *P*_*o*_ is the observed agreement and *P*_*e*_ is the expected agreement by chance. The difference between the two metrics is that the Fleiss’ kappa calculates *P*_*e*_as the expected value of the class distribution, whereas the S score calculates *P*_*e*_ as the inverse of the number of classes. The S score is more robust when there is an imbalance between the two possible responses (yes or no). The values of these two metrics were interpreted using the reference ranges presented in Table 2. Additionally, for each of the thirteen shared sets, we calculated the percentage of marketing instances in which all three children agreed.

**Table 2:** Fleiss’ Kappa and S score interpretations

κ or S	Interpretation
≤ 0·01	Poor agreement
0·01–0·20	Slight agreement
0·21–0·40	Fair agreement
0·41–0·60	Moderate agreement
0·61–0·80	Substantial agreement
0·81–1·00	Almost perfect agreement

### Data pre-processing

For the variables collected with the questionnaire, we treated household education, household income, perceived income adequacy, frequency of screen use and dietary intake data (i.e. frequency of consuming specific types of food) as ordinal variables. Sex and ethnicity were treated as nominal categorical variables. Age, years lived in Canada, weight and height were used as continuous variables. Weight and height were used to calculate BMI z-scores using WHO guidelines^(38)^ which was also included as a continuous variable.

### Extracting elements contained in the food marketing instances

Elements contained in the food marketing instances were extracted using Google Vision API^(39)^, a commercial machine learning-based service for image processing. For each of the 1365 food ads, we used Google Vision API to extract the following four elements:

1. Text: Letters and words embedded in the image using optical character recognition methods.
2. Labels: General themes or topics associated with the image, such as locations, activities, animal species and products.
3. Objects: Persons, animals or items included in the image.
4. Logos: Popular product or brand logos within the image.

**Fig. 2:**
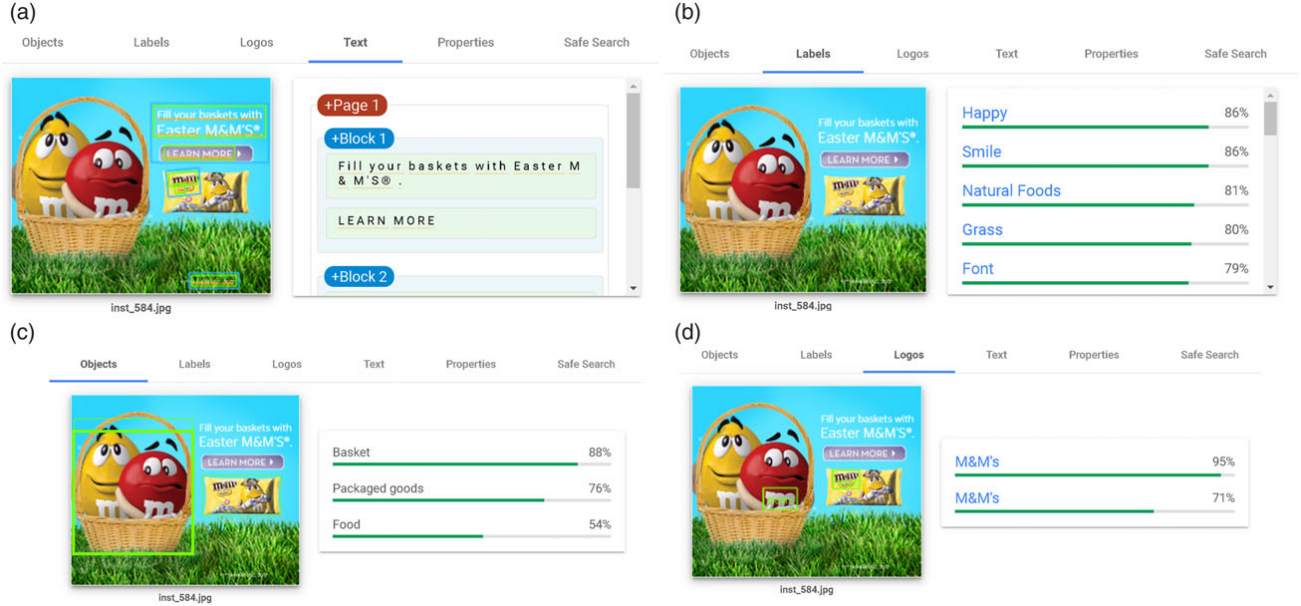
Food ad elements extracted using Google Vision API. The four extracted elements were: (a) text, (b) labels, (c) objects, and (d) logos

Figure 2 illustrates the identification of text, labels, objects and logos for one of the food marketing instances that were shown to participants. For each element, Google Vision API detected none, one or multiple items. For instance, panel (b) in Fig. 2 shows that Google Vision API identified happy, smile, natural foods, grass and font as labels for that food ad.

This procedure resulted in a dataset composed of 1365 rows (one per food ad) and four columns (one per element). Each cell of this dataset corresponds to the text, labels, objects or logos of the food ad.

### Constructing the final dataset

**Fig. 3:**
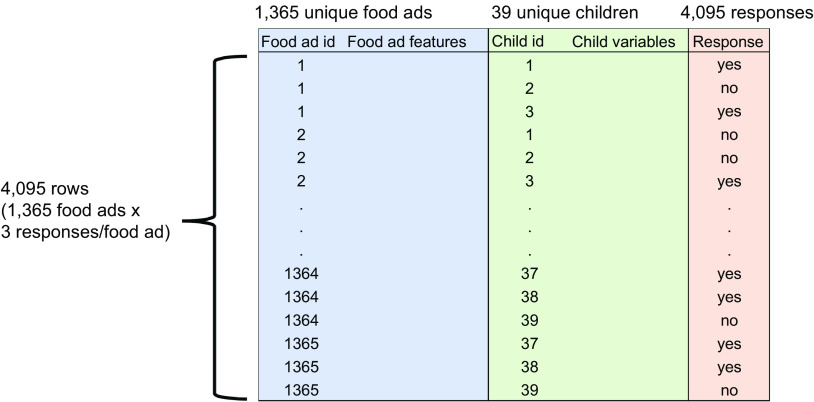
Format of the final dataset. Each row contains information related to the food ad elements, children’s variables, and their response for that food marketing instance

Because three children evaluated each food ad, each food ad received three responses. Consequently, a total of 4095 responses from thirty-nine children were collected in this work. We used the 4095 responses, the food ad elements and the children’s variables collected with the questionnaire to build a final dataset. Figure 3 shows the format of the final dataset. Each row in the final data set contains food ad elements, children’s variables and their response to that food instance. The dataset contains 1365 unique food ads, thirty-nine different children and a total of 4095 responses.

### Machine learning approach

We used a machine learning approach to predict whether the food marketing instances appealed to children. To train and test our machine learning models, we split the final dataset (see Fig. 3) by randomly assigning twelve out of thirteen shared sets to the training set and the remaining one to the test set. Since each set was evaluated by three independent children, the training set in total had thirty-six children, and the test set only had three. The training set was used to build the prediction models, whereas the test set was used to calculate the performance of the trained models. As we split the data based on the shared sets, none of the food ads were included in both the training and test sets. This helped to ensure that the features of the food ads did not bias model performance.

Machine learning models relate a set of predictors to an outcome or response variable. For our study, the response variable was the answer (yes or no) given by each child when they viewed the food marketing instances. The predictors were of two types. The first type of predictor was children’s socio-demographic characteristics, weight, height, BMI z-score, frequency of screen use and dietary intake variables. The second type of predictors were the text, logos, objects and labels extracted from the food marketing instances using Google Vision API.

**Fig. 4:**
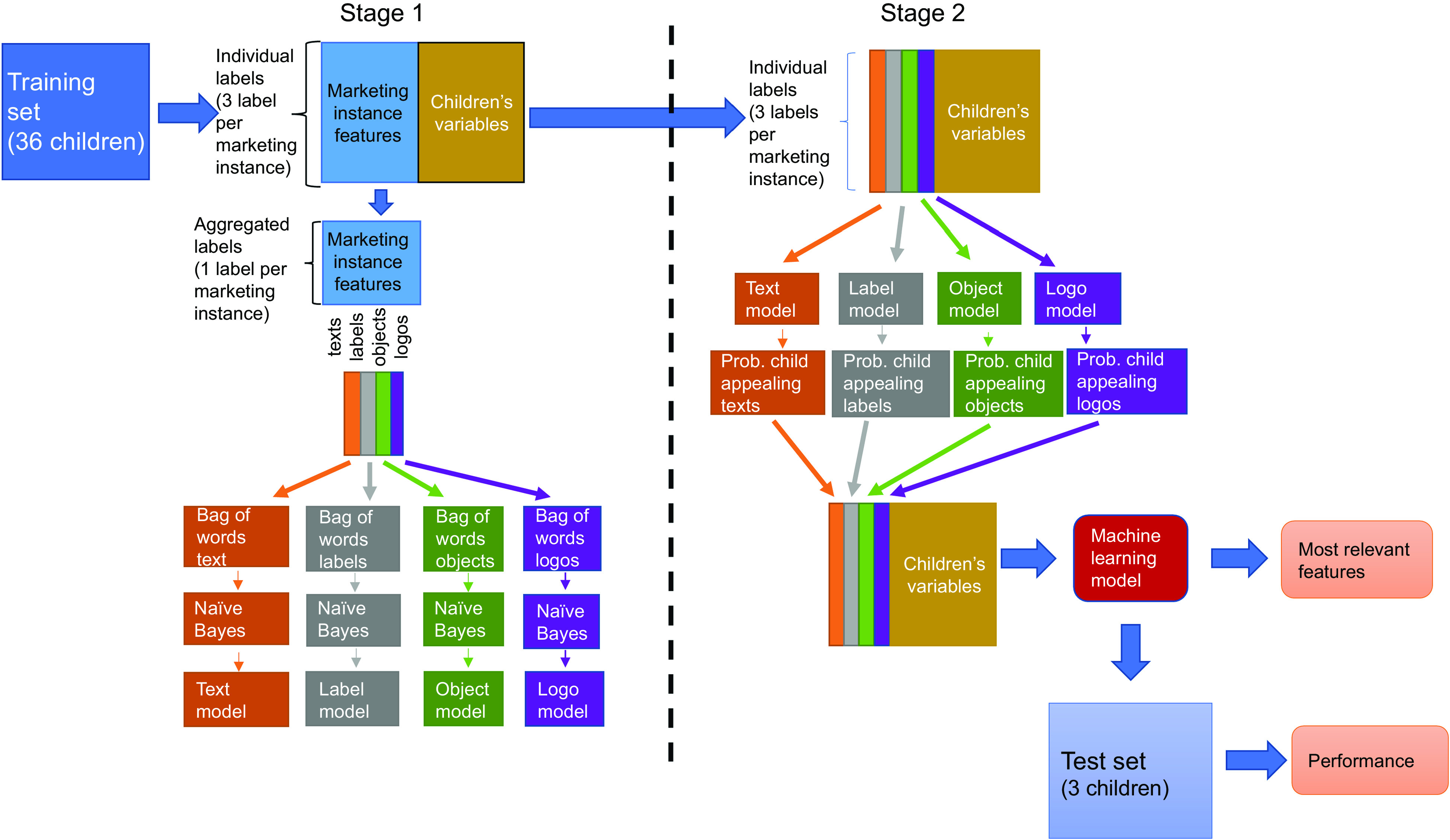
Machine learning approach for predicting child appealing food marketing instances. The approach comprised two stages. In the first stage, text, logos, labels, and objects contained in the food marketing instances were used to calculate the probability of the instance being child appealing given the text, logos, labels, and objects. In the second stage, the output from the first stage was combined with children's sociodemographic characteristics, weight, height, standardized BMI, frequency of screen use, and dietary intake variables to predict whether the food marketing instances appealed to children using logistic regression, random forest, gradient boosting trees, and conditional inference tree models

Figure 4 shows the machine learning approach, which was composed of two stages to predict whether the food marketing instances appealed to children. The first stage dealt with processing the predictors extracted from the food marketing instances, whereas the second stage combined the output from the first stage with children’s socio-demographic characteristics, weight, height, standardised BMI, frequency of screen use and dietary intake variables to predict whether the food marketing instances were child appealing using logistic regression, random forest, gradient boosting trees and conditional inference tree models.

#### Stage 1: Processing features from the food marketing instances

The goal of the first stage was to estimate the probability that a given food marketing instance appealed to children, given the elements (text, labels, objects and logos) contained in it. This stage only considered food ad elements and not children’s variables.

Because each food marketing instance was evaluated by three children, the information related to each marketing instance was summarised in three rows of the training dataset, each one corresponding to the response of one child. To increase confidence that a given ad did or did not appeal to children, we aggregated the three children’s responses for each ad by taking the mode. The mode ensured that the most frequent response was kept. For instance, if two children responded ‘yes’ when asked whether the food ad was appealing to them, and one child answered ‘no’, the aggregate response for the food ad was yes (child appealing).

##### Calculating the probability that a marketing instance appealed to children, given its elements

After aggregating children’s responses, the number of rows in the training set was reduced by a factor of three. This new aggregated training set was used to build four models to calculate the probability that a marketing instance appealed to children. Specifically, the four models corresponded to the probability that an ad appealed to children based on text, labels, objects and logos, respectively.

To build each of these four models, we used a natural language processing technique called bag of words. A bag of words represents a set of documents as a set (‘bag’) of its words. In our case, the food ads were the documents, and the different elements were the corresponding words (text, labels, objects or logos). The first step in a bag of word approach is to identify the unique words across all the documents. Therefore, we identified the unique words provided by Google Vision API across the food ads. Once the unique words are identified, the second step is to generate a matrix by placing each unique word as columns and each document as rows. The intersection of each document and word is the count of how many times the word is mentioned in that document. Thus, in our case, the cells of this matrix showed how many times each word (text, label, object or logo) was mentioned in a food marketing instance.

The last step was to use the bag of words matrix with the aggregated children’s responses to build the model. To this aim, we used a multinomial Naïve Bayes model. Naïve Bayes is a model that uses the Bayes theorem to calculate for each sample in the dataset the likelihood of belonging to each class. In our case, the Naïve Bayes calculated for each food ad its likelihood of being child appealing or not based on the text, labels, objects and logos encoded in the bag of words matrix.

#### Stage 2: Training machine learning models to predict whether food marketing appealed to children

The text, labels, objects and logos Naïve Bayes models trained in Stage 1 were used to calculate the probability that a marketing instance appealed to children. Each Naïve Bayes model produced a probability value, having in total four probabilities for each food marketing instance. These four probabilities were combined with children’s socio-demographic characteristics, weight, height, standardised BMI, frequency of screen use and dietary intake variables to predict whether the food marketing instance appealed to children using four different machine learning models: logistic regression, random forest, gradient boosting trees and conditional inference tree. The logistic regression, random forest and gradient boosting tree were implemented using the *statsmodels* ^(40)^ and *sklearn* ^(41)^ libraries in Python. The conditional inference tree was implemented using the *party* ^(42)^ package in R.

Logistic regression is a statistical model that calculates the probability of an event occurring based on a set of independent predictors^(43)^. In our case, the event was whether a food marketing instance appealed to children, whereas the independent predictors were the four probabilities for the text, logos, objects and labels calculated during Stage 1 and children’s socio-demographic characteristics, weight, height, standardised BMI, frequency of screen use and dietary intake variables. Logistic regression performs multiple iterations to estimate coefficients for the independent predictors that best fit with the event occurrence. These coefficients can be transformed to estimate odds ratios. The OR represent the odds that the event would occur based on the independent predictors’ values.

Random forest and gradient-boosting trees are machine-learning predictive models composed of multiple decision trees^(44)^. A decision tree is a tree-like model composed of nodes, branches and leaves. Each node uses one of the independent predictors to ask a question, the branches represent the possible answers to that question and the leaves represent the predicted class^(45)^. Building a decision tree requires selecting the best independent predictors for each node to split the data in a manner that best differentiates between the two classes of interest (i.e. child appealing *v*. not child appealing). Random forest and gradient-boosting trees use the Gini index to measure class impurity after each split, selecting the predictor that minimises class impurity. The difference between random forest and gradient-boosting trees is how the multiple decision trees are created and aggregated. Random forest uses a technique called bagging in which each decision tree is built independently, whereas, in gradient-boosting trees, the decision trees are added iteratively, aiming to improve deficiencies in previous trees^(46)^. This iterative process is controlled by a learning rate, which defines the contribution of each tree to the prediction of the outcome.

The conditional inference tree is composed of only one decision tree. The difference is that this decision tree is constructed by using a hypothesis significance test to select the best independent predictor at each node^(47)^. Thus, at each node, the algorithm tests, for each possible predictor, the null hypothesis between that predictor and the response variable (i.e. child appealing *v*. not child appealing). Then, the algorithm selects the predictor with the lowest *P*-value to split the node into two new nodes. The algorithm repeats this process until no further splits can be made while respecting the minimum number of observations in terminal nodes.

To reduce overfitting, the logistic regression model was trained using ridge regularisation (L2-norm). Similarly, the random forest, gradient-boosting tree and conditional inference trees were limited to a maximum depth of five levels to avoid complex models leading to overfitting. The model’s parameters were tuned using Bayesian optimisation on the training set using the search space presented in Table 3.

**Table 3:** Bayesian optimisation search space for the machine learning model parameters

Model	Parameter	Search space			
		Lower limit	Upper limit	Type	Scale
Logistic regression	C (regularisation strength)	0·02	1	Decimal	Logarithmic
Random forest	Number of estimators	100	5000	Integer	Linear
	Maximum depth	1	3	Integer	Linear
	Minimum samples leaf	200	500	Integer	Linear
	Maximum features	2	5	Integer	Linear
Gradient-boosting tree	Number of estimators	300	800	Integer	Linear
	Learning rate	0·001	0·01	Float	Logarithmic
	Maximum features	2	5	Decimal	Linear
	Maximum depth	1	4	Decimal	Linear

### Performance of the models

The best parameters were used to retrain each model using all training data, and the performance of the model was calculated on the held-out test set using the area under the receiver operating characteristic curve (ROC-AUC), the true-positive rate and the true negative rate. The true positive rate was calculated as the number of child-appealing food marketing instances correctly classified divided by the total number of child-appealing food marketing instances. Likewise, the true negative rate was calculated as the number of non-child appealing food marketing instances correctly classified divided by the total number of non-child appealing food marketing instances.

### Relevant predictors

For the logistic regression, OR, 95 % CI and *P*-values were calculated for each predictor variable. Statistical significance was set at *P* < 0·05. For the ensemble models (random forest and gradient-boosting trees), feature importance was determined using permutation importance. Permutation importance was determined by comparing the score calculated using the original dataset with the score obtained by permuting the elements of each column. The features were ranked based on the increase in error due to the permutation. Finally, the conditional decision tree enabled us to visualise the features that were most important in determining whether marketing instances appealed to children or not.

### Assessing the impact of changing the question

Since eighteen children answered the question ‘Is this ad for kids your age?’ and twenty-one children answered, ‘Is this ad for kids like you?’, we repeated the machine learning approach for logistic regression with the question represented as a binary predictor variable. Our rationale was to investigate if the impact of changing the question was significant.

## Results

### Descriptive statistics

Table 4 provides descriptive statistics for the numerical and categorical variables of the thirty-nine children. The average age was 9·1 years, and the number of males was similar to the number of females. Almost half of the children were White and came from households where a parent had received a bachelor’s degree or higher and had an annual household income > CAD $ 100 000. Most children reported spending at most 9 hours per week watching television, using the internet, and consumed unhealthy snack foods (e.g. sodas, chips and candy) four times per week or less. Most children consumed vegetables and fruits at least once per day.

**Table 4:** Descriptive statistics for the thirty-nine participants

Numerical variable	Mean	sd
Age (years)	9·1	1·8
Years lived in Canada	8·9	2·7
Weight (kg)	31·1	9·8
Height (cm)	134·7	13·2
BMI z-score	−0·3	1·6
Age (years)	9·1	1·8
Categorical variable	Count	Percentage (%)
Sex		
Male	18	46·2
Female	21	53·8
Household education*		
≤ High school	1	2·6
High school	3	7·7
Trade certificate or diploma from a vocational school or apprenticeship training	1	2·6
University certificate below Bachelor’s level	5	12·8
Bachelor’s degree	17	43·6
University degree or certificate above Bachelor’s degree	12	30·8
Don’t know/decline to answer	0	0
Household income		
≤ $20 000	1	2·6
$20 000–$344 999	0	0
$35 000–$49 999	2	5·1
$50 000–74 999	7	17·9
$75 000–$99 999	9	23·1
≥ $100 000	20	51·3
Don’t know/decline to answer	0	0
Perceived income adequacy^†^		
Very difficult	0	0
Difficult	3	7·7
Neither easy nor difficult	16	41·0
Easy	12	30·8
Very easy	7	17·9
Don’t know/decline to answer	1	2·6
Ethnicity		
Indigenous	0	0
Middle East	0	0
White	19	48·7
Asian	13	33·3
South Asian	1	2·6
Black	1	2·6
Latin American	2	5·1
Don’t know/decline to answer	3	7·7
Weekly hours of television		
Do not watch television	4	10·3
≤ 4 h	12	30·8
4–9 h	12	30·8
10–15 h	5	12·8
≥ 15 h	6	15·4
Weekly hours of internet		
Do not use internet	3	7·7
≤ 4 h	11	28·2
4–9 h	13	33·3
10–15 h	5	12·8
≥ 15 h	7	17·9
Weekly hours of smartphone		
Do not use smartphone	35	89·7
≤ 4 h	3	7·7
4–9 h	1	2·6
10–15 h	0	0
≥ 15 h	0	0
Weekly hours of tablet/computer		
Do not use tablet or computer	5	12·8
≤ 4 h	11	28·2
4–9 h	8	20·5
10–15 h	7	17·9
≥ 15 h	8	20·5
Sodas per week		
< 1 per week	13	33·3
Once per week	8	20·5
2–4 times per week	8	20·5
5–6 times per week	4	10·3
Once a day	4	10·3
2–3 times per day	0	0
≥ 4 times per day	2	5·1
Chips per week		
< 1 per week	4	10·3
Once per week	6	15·4
2–4 times per week	23	59·0
5–6 times per week	3	7·7
Once a day	3	7·7
2–3 times per day	0	0
≥ 4 times per day	0	0
Candy/chocolate per week		
No answer	1	2·6
< 1 per week	1	2·6
Once per week	7	17·9
2–4 times per week	16	41·0
5–6 times per week	6	15·4
Once a day	5	12·8
2–3 times per day	3	7·7
≥ 4 times per day	0	0
Vegetables per week		
< 1 per week	1	2·6
Once per week	2	5·1
2–4 times per week	5	12·8
5–6 times per week	1	2·6
Once a day	13	33·3
2–3 times per day	17	43·6
≥ 4 times per day	0	0
Fruits per week		
< 1 per week	0	0
Once per week	1	2·6
2–4 times per week	4	10·3
5–6 times per week	2	5·1
Once a day	14	35·9
2–3 times per day	17	43·6
≥ 4 times per day	1	2·6

*Corresponds to highest level attained by any parent in the household.

† Corresponds to how difficult or easy it is for the household to make ends meet.

### Child agreement

Table 5 shows agreement between children on whether the food marketing instances appealed to them. Using Fleiss’ kappa statistic, only one out of thirteen shared sets achieved fair agreement. Based on the S score, four shared sets had a fair agreement. The percentage of sets in which all three children agreed ranged between 8 and 53 %.

**Table 5:** Child agreement using the Fleiss’ kappa, the S score and the total percentage of cases in which all three children provided the same answer for food marketing instances

Sets	Number of marketing instances	Fleiss’ kappa	Fleiss’ kappa interpretation	S score	Bennett score’s interpretation	Perfect agreement (%)
1–2	72	−0·01	Poor agreement	0·00	Poor agreement	25
3–4	102	−0·28	Poor agreement	−0·23	Poor agreement	8
5–6	102	−0·01	Poor agreement	−0·01	Poor agreement	25
7–8	104	−0·03	Poor agreement	0·35	Fair agreement	52
9–10	104	−0·03	Poor agreement	0·01	Slight agreement	26
11–12	104	−0·11	Poor agreement	0·19	Slight agreement	39
13–14	104	−0·24	Poor agreement	−0·22	Poor agreement	8
15–16	104	0·09	Slight agreement	0·10	Slight agreement	33
17–18	104	0·02	Slight agreement	0·03	Slight agreement	27
19–20	104	0·05	Slight agreement	0·23	Fair agreement	53
21–22	114	0·08	Slight agreement	0·23	Fair agreement	42
23–24	124	0·21	Fair agreement	0·25	Fair agreement	44
25–26	124	0·07	Slight agreement	0·10	Slight agreement	32

### Model performance

The logistic regression, random forest, gradient boosting tree and conditional inference tree models achieved ROC-AUC of 0·61, 0·65, 0·60, and 0·60, respectively (Table 6). True positive rates tended to be slightly higher than true negative rates. The random forest was the model with the best performance, yielding a ROC-AUC of 0·65.

**Table 6:** Performance of the machine learning models for predicting whether food marketing instances appealed to children

Model	True positive rate	True negative rate	ROC-AUC
Logistic regression	57·7	57·6	0·61
Random forest	60·7	56·5	0·65
Gradient boosting	59·2	55·9	0·60
Conditional inference tree	59·0	60·0	0·60

ROC-AUC: The receiver-operating characteristic curve and AUC.

### Logistic regression model

Table 7 shows the odds ratios from the logistic regression model. The logistic regression yielded five significant variables. The probabilities of the food marketing instance being child appealing, given its text and logos were significant. An increment of 1 % on text and logo probabilities increased the odds of a child finding a food marketing instance appealing by 2 % and 1 %, respectively. Vegetable consumption was also significant; children consuming vegetables more frequently found the food marketing instances less appealing. Males had 21 % lower odds of finding the food marketing instances appealing than females, and Asian children had significantly higher odds of finding instances appealing than White children.

**Table 7:** OR, 95 % CI and *P*-values for the logistic regression model to predict whether food marketing instances appealed to children

Group	Variables	OR	95 % CI	*P* value
Food marketing instance attributes	Text*	1·02	1·01, 1·02	< 0·001
	Labels	1·00	0·99, 1·01	0·963
	Objects	1·01	1·00, 1·01	0·045
	Logos*	1·01	1·00, 1·01	0·002
Children’s characteristics	Age	0·98	0·88, 1·09	0·699
	Years living in Canada	0·99	0·93, 1·05	0·744
	Weight	1·00	0·97, 1·03	0·902
	Height	1·00	0·98, 1·02	0·955
	BMI z-score	0·98	0·88, 1·10	0·790
Children’s household characteristics	Household education	1·04	0·97, 1·12	0·313
	Household income	1·02	0·88, 1·20	0·762
	Perceived income adequacy	0·94	0·80, 1·11	0·476
Frequency of screen use	Weekly hours of television	1·09	0·98, 1·20	0·113
	Weekly hours of internet	0·91	0·77, 1·08	0·290
	Weekly hours of smartphone	1·01	0·73, 1·39	0·945
	Weekly hours of tablet/computer	0·90	0·80, 1·01	0·076
Dietary intake	Sodas per week	0·93	0·83, 1·05	0·249
	Chips per week	1·06	0·93, 1·21	0·356
	Candy/chocolate per week	1·00	0·92, 1·08	0·961
	Vegetables per week*	0·89	0·83, 0·95	0·001
	Fruits per week	0·97	0·89, 1·07	0·574
Sex	Male (female as reference)*	0·79	0·63, 0·98	0·035
Ethnicity	Asian (White as reference)*	1·36	1·07, 1·72	0·011
	Black (White as reference)	0·83	0·41, 1·67	0·594
	Latin American (White as reference)	0·83	0·41, 1·67	0·594
	South Asian (White as reference)	0·77	0·35, 1·69	0·513

*Indicates that the variable is statistically significant with an alpha set at 0·05.

### Assessing the impact of changing the question

The question variable was NS, indicating that changing the question did not have any significant effect on predicting whether the food marketing instances appealed to children. Moreover, OR for all predictors were similar to those obtained when question id was not included as a predictor (see Appendix 2).

### Ensemble models

**Fig. 5:**
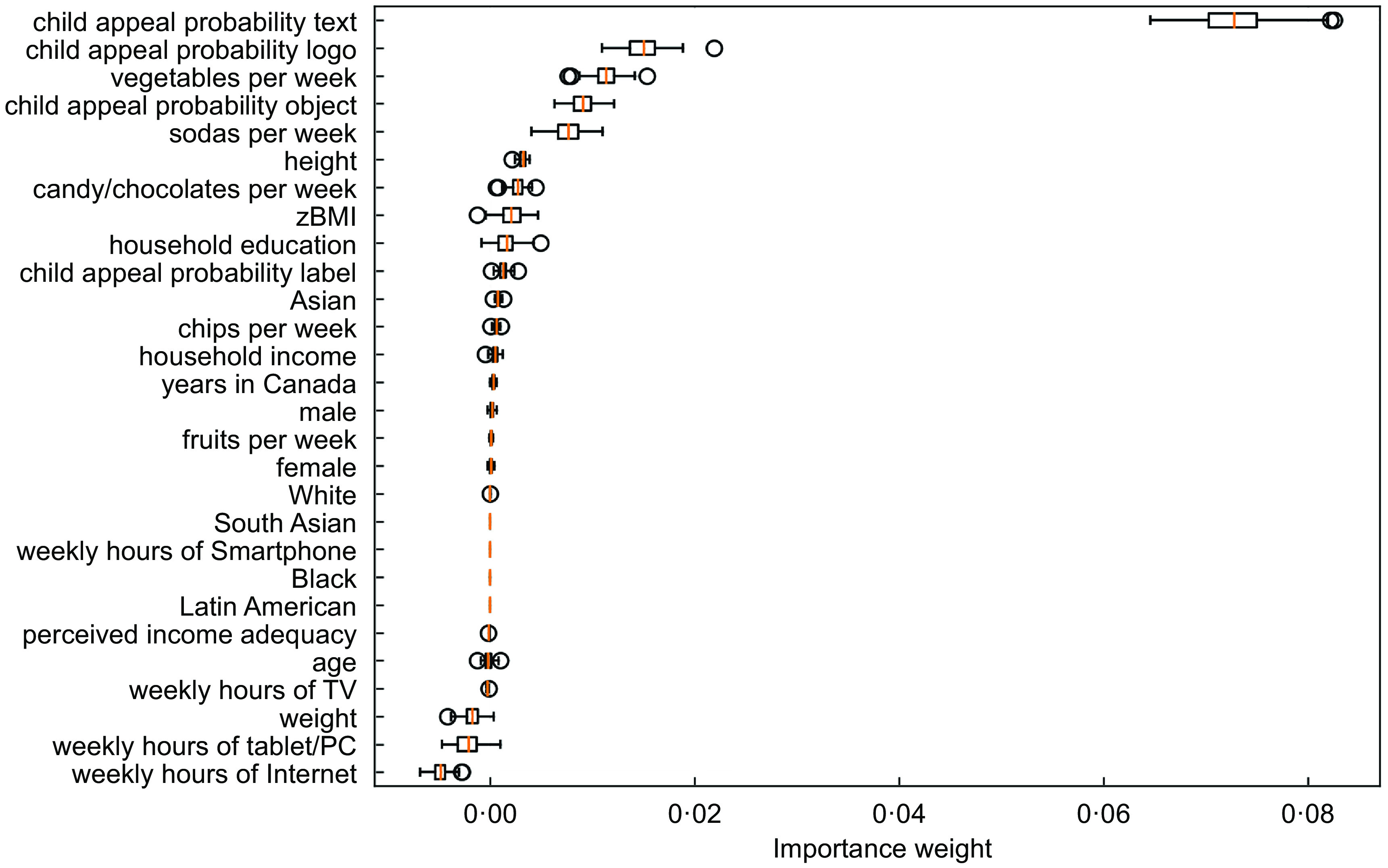
Variable importance to predict whether food marketing instances appealed to children in the random forest model

**Fig. 6:**
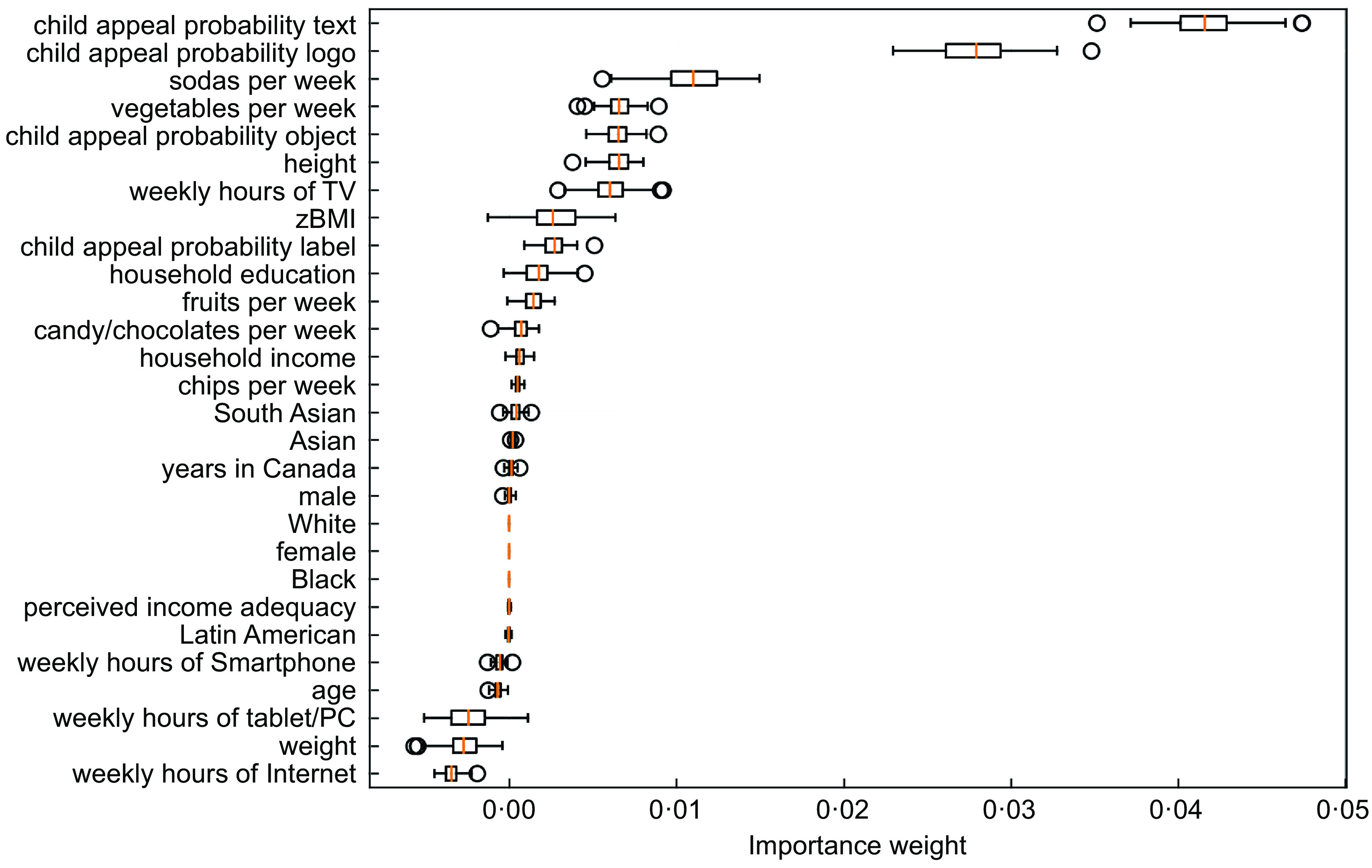
Variable importance to predict whether food marketing instances appealed to children in the gradient boosting tree model

Figures 5 and 6 show the feature importance provided by the random forest and gradient-boosting trees. Marketing features in the text, logos and objects contained in the food marketing instances were the most important predictors of whether food marketing instances appealed to children. In both models, weekly consumption of vegetables and soda was also important. The models also underscored the importance of height and BMI z-score. For the frequency of screen use variables, the gradient-boosting model selected weekly hours spent on television as an important predictor.

### Conditional inference tree

**Fig. 7:**
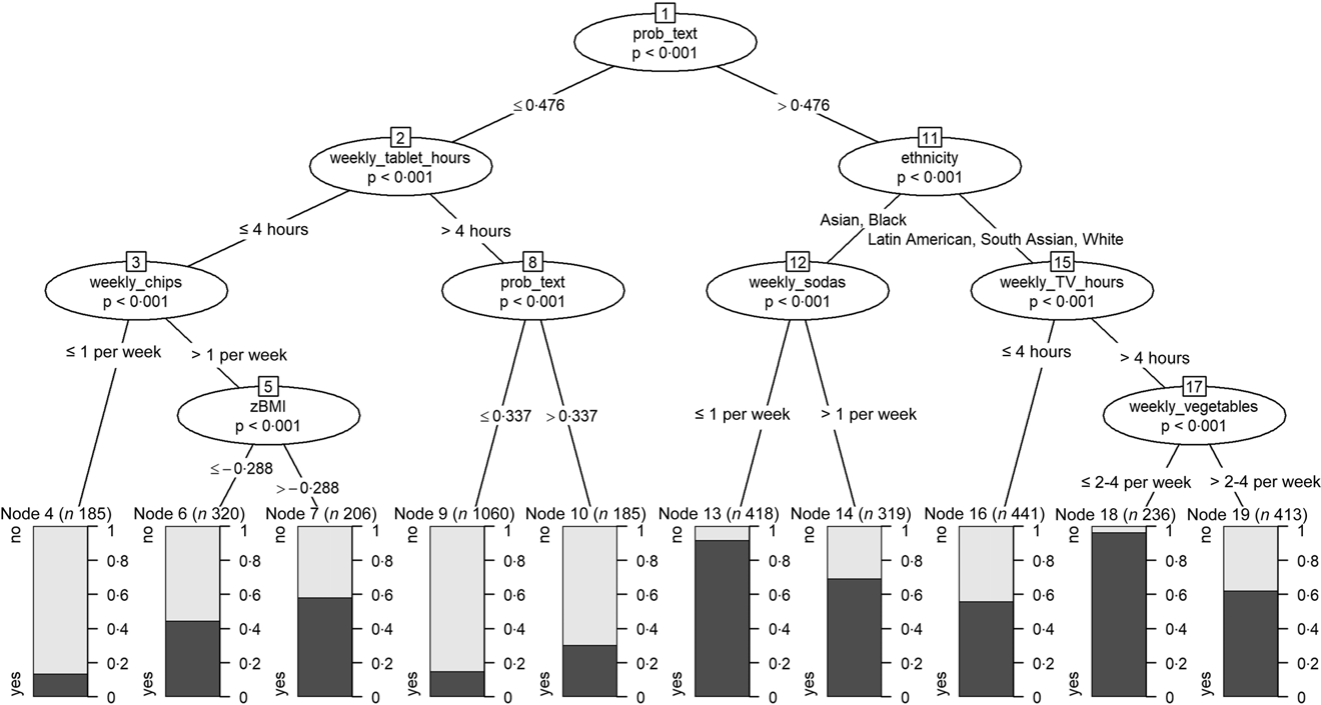
Conditional inference tree for predicting whether food marketing instances appealed to children

Figure 7 shows the conditional inference tree when its maximum height is constrained to five levels. The text was again the most important factor in predicting whether marketing appealed to children. The node with the highest proportion of child-appealing food marketing instances (97 %; Node 18; *n* 236) was for Latin American, South Asian and White children, who watched television more than 4 hours per week and consumed vegetables less than times per week. The node with the lowest proportion of positive responses (14 %; Node 4; *n* 185) was for children who used tablets less than 4 hours per week and consumed chips at most once weekly. Notably, the two branches suggest that children with higher consumption of chips or lower consumption of vegetables found more food marketing instances appealing.

## Discussion

The most important predictors of child appeal were the elements contained in the food marketing instances, particularly the embedded text and logos. Other important predictors of the appeal of marketing included children’s consumption of vegetables and soda, children’s sex and weekly television hours.

Children’s weekly consumption of vegetables was an important determinant of whether they found food marketing to be appealing. This predictor was deemed important by all models, whereby children with low consumption of vegetables found more food marketing instances to be appealing. The random forest and gradient boosting tree also indicated the relevance of soda consumption, thus again showing that dietary intake predicts the appeal of food marketing. The conditional inference tree indicated that children who watched television more often found more food marketing instances to be appealing. Importantly, however, the random forest and gradient-boosting tree showed that the probability of a food marketing instance being child appealing given its text and logos was a much stronger predictor of child appeal than any of the children’s variables (i.e. socio-demographic characteristics, weight, height, BMI, frequency of screen use, dietary intake).

Children’s sex and ethnicity were also crucial in predicting whether food marketing appealed to them. Female children found more food marketing instances to be appealing than their male peers, suggesting that females were more receptive to food marketing strategies. Asian children had higher odds of identifying instances as appealing than White children based on the logistic regression model. There were no significant differences for children of other ethnicities. However, we note that most of the children (32 out of 39) were White or Asian, thereby it is challenging to make any firm conclusions in this respect.

We also found substantial disagreement among children as to which digital food marketing appealed to them. The low agreement between the children, based on Kappa’s Fleiss and S scores, shows that a marketing strategy can appeal to one child but not to another despite the fact that they may have similar characteristics (e.g. age, sex, household income). This disagreement shows the ambiguity of determining whether a food ad appeals to children. It also highlights how challenging it is to regulate digital food marketing as it is unclear which marketing techniques should be prohibited.

Because the text and logos contained in the food ads are related to promotional messages and well-known food brand characters (e.g. Ronald McDonald, Tony the Tiger), the relevance of these predictors given by the machine learning models shows their capacity to persuade children. This finding is consistent with previous studies which have reported that spokes characters or messages related to taste, fun or nutrition are among the most persuasive marketing strategies for children^(12)^.

Our finding that television time was an important predictor of whether children found food marketing to be appealing is consistent with previous studies that have reported that children’s food choice is affected by exposure to television^(18,20)^. Thus, this supports the necessity of regulations to restrict the marketing of unhealthy food to children on television.

Previous research has underscored the challenges of operationalising what constitutes ‘child appealing’ food marketing^(9)^. Our study did not overcome that challenge, but it can provide some insights in that direction. Specifically, given the high relevance of text and logos embedded in food ads as predictors of child appeal, it will be crucial to focus on these marketing elements when designing regulations to limit food marketing to children due to their persuasive effect on children.

Our work also supports the call for understanding how children’s backgrounds influence their attraction to food ads since marketing techniques do not have the same effect on all children^(12)^. Although we did not use qualitative techniques to understand the reason behind children’s answers, our quantitative approach showed how several characteristics of children predict whether digital food marketing appeals to them. For instance, we found that low consumption of vegetables can increase the likelihood that children find food ads to be appealing.

### Strengths and limitations

To the best of our knowledge, this is the first study to use machine learning to quantify how marketing instance features and children’s socio-demographic characteristics, weight, height, BMI, frequency of screen use and dietary intake are associated with whether digital food marketing appeals to children. Our findings provide key data to understand which factors shape the appeal of digital food marketing to children.

Although the fitted models were able to identify important determinants of child appeal, model performance was poor (around 60 % in all metrics). One reason that can explain the limited performance of the models was the low agreement between children as to which marketing instances appealed to them. Low inter-rater agreement (children in our case) negatively impacts the performance of machine learning models in their attempt to generalise from training data^(48,49)^. Thus, the low inter-rate agreement may have affected the capacity of our models to identify patterns explaining whether food marketing appealed to children. Another reason might be that additional predictor variables from the children need to be included to strengthen the predictive capacity of the models.

We did not use filtering techniques to remove food ads with low agreement because, due to the nature of this work, there is no objective truth (i.e. actual class: child appealing *v*. not child appealing) for each food ad. As such, we were unable to detect which children were systematically providing inconsistent answers. Moreover, filtering is often discouraged for subjective classification tasks, such as those in our work, because they can yield unrealistically high-performance scores for tasks in which genuine differences in interpretation are valid^(50,51)^.

Most of the children who participated in this study came from relatively advantaged households in terms of income and level of education, and most children were White or Asian. Our sample size was also small, although children labelled a relatively large number of marketing instances. Our results may therefore have limited generalisability. However, it is notable that despite the similarity of children in our sample, there was a substantial lack of agreement as to which food marketing instances appealed to them, suggesting that disagreement may have been even greater had our sample been more diverse.

In this study, we did not consider sex or age when assigning children to sets. This may have contributed to the low agreement among children, as children of similar age and sex might be attracted by similar marketing instances. Future studies should analyse whether grouping children by these characteristics and showing them food marketing instances that specifically target these groups (e.g. males 6–9 years *v*. females 6–9 years) improves agreement. We also note that we did not control for prior exposure to food marketing. As awareness and familiarity with food products and brands can influence preferences, prior exposure may have contributed to differences in children’s responses and should be considered in future studies. Finally, children in the current study were shown ads for both healthy and unhealthy products. Future studies should explore whether predictors of child appeal differ for healthy and unhealthy food ads.

## Conclusion

This study examined associations between marketing instance features and children’s socio-demographic characteristics, weight, height, BMI, frequency of screen use and dietary intake to investigate the most important predictors of whether digital food marketing appeals to children. There was low agreement among children as to which food marketing instances appealed to them. Text and logos embedded in the food marketing instances were the most important predictors of child appeal. Children’s consumption of vegetables and soda, sex and weekly hours of television were also important predictors of the appeal of food marketing.

## Appendix 1 Companies and brands featured in food marketing ads

**Table:**

Food category	Brand	Company
Almond milk	Almond breeze	Blue Diamond Growers
Almond milk	Silk	Danone
Breakfast cereal	Cheerios	Nestle
Breakfast cereal	Chex mix	General Mills
Breakfast cereal	Lucky charms	General Mills
Breakfast cereal	Toast crunch	General Mills
Breakfast cereal	Kashi cereal	Kellogg’s
Breakfast cereal	Froot loops	Kellogg’s
Breakfast cereal	Frosted flakes	Kellogg’s
Candy	Airheads gum	Perfetti Van Melle
Candy	5 gum	Wrigley Company
Candy	Sour punch	American Licorice Company
Candy	Fruit by the foot	General Mills
Candy	Haribo goldbears	Haribo
Candy	Sweetarts	Nestle
Candy	Mentos chewy mints	Perfetti Van Melle
Candy	Mentos	Perfetti Van Melle
Candy	Ice breakers	The Hershey Company
Candy	Jolly rancher	The Hershey Company
Candy	Twizzlers	The Hershey Company
Canned beans	Green giant	General Mills
Canned soup	Well yes!	Campbell Soup Company
Canned soup	Campbell’s tomato soup	Campbell Soup Company
Cheese	Cracker barrel cheese	Kraft Heinz Company
Cheese	Kraft singles	Kraft Heinz Company
Cheese	Go veggie cheese	GreenSpace Brands
Chips	Pringles	Kellogg’s
Chips	Cheetos	PepsiCo
Chips	Frito lay	PepsiCo
Chips	Miss Vickie’s	PepsiCo
Chips	Ruffles	PepsiCo
Chips	Stacy’s	PepsiCo
Chips	SunChips	PepsiCo
Chips	Tostitos	PepsiCo
Chips	Skinny pop	The Hershey Company
Chocolate	Kinder joy	Ferrero International S.A
Chocolate	Lindt	Chocoladefabriken Lindt & Sprungli
Chocolate	Cadbury	Mondelez International Inc
Chocolate	Nutella	Ferrero International S.A
Chocolate	Ferrero rocher	Ferrero International S.A
Chocolate	Skittles	Mars, Incorporated
Chocolate	Snickers	Mars, Incorporated
Chocolate	Twix	Mars, Incorporated
Chocolate	Milka	Mondelez International Inc
Chocolate	Toblerone	Mondelez International Inc
Chocolate	Kit Kat	Nestle
Chocolate	M&M’s	Mars, Incorporated
Chocolate	Reese’s	The Hershey Company
Chocolate beverage	TruMoo	Dean Foods
Chocolate beverage	Nesquik	Nestle
Coffee shop	Starbucks	
Coffee shop	Tim Hortons	
Cookies	Chips ahoy!	Mondelez International Inc
Cookies	Christie peek freans	Mondelez International Inc
Cookies	Keebler	Kellogg’s
Cookies	Oreo	Mondelez International Inc
Cookies	Chewy dipps	PepsiCo
Cookies	WhoNu?	Suncore Products, L.L.C
Cookies	Voortman cookies	Voortman Cookies Limited
Crackers	Triscuit	Mondelez International Inc
Crackers	Cheez it	Kellogg’s
Crackers	Club crackers	Kellogg’s
Crackers	Mary’s crackers	Kameda USA, Inc
Crackers	Ritz	Mondelez International Inc
Eggs	Get cracking	Egg Farmers of Ontario
Energy drink	Monster energy	The Coca-Cola Company
Energy drink	Red bull	Red Bull GmbH
Fast food	AWG	
Fast food	Arby’s	
Fast food	Burger king	
Fast food	Chipotle	
Fast food	Dairy queen	
Fast food	Domino’s	
Fast food	KFC	
Fast food	McDonald’s	
Fast food	Pizza hut	
Fast food	Subway	
Fast food	Taco bell	
Fast food	Taco time	
Fast food	Wendy’s	
Fast food	Boston pizza	
Fast food	Pita pit	
Frozen food	Barber foods stuffed chicken breast	Barber Foods
Fruit	Dole diced peaches	PepsiCo
Fruit	Chiquita banana	Chiquita Brands International
Fruit	Washington apples	International Farming Corporation
Fruit	Pure gold Pineapples	Fullerton Farms
Fruit	Stemitt world famous fruit apples	The Mathison family
Fruit	Sun-Maid	Sun-Maid Growers of California
Ice cream	Drumstick	Nestle
Ice cream	Ben & Jerry’s	Unilever
Ice cream	Klondike	Unilever
Ice cream	Magnum almond	Unilever
Ice cream	Talenti gelato	Unilever
Ice cream	So delicious	WhiteWave Foods Company
Meat	Tyson	Tyson Foods
Meat	Beyond burger	Beyond Meat
Milk	Fairlife	The Coca-Cola Company
Milk	Horizon organic	Danone
Milk	Dairy pure	Dean Foods
Milk	Lactaid	Johnson & Johnson Company
Oats	Quaker	PepsiCo
Organic produce	Misfits market	Abhi Ramesh - Founder
Peanut butter	Jif peanut butter	The J.M. Smucker Company
Salad	Fresh express	Chiquita Brands International
Sauce	Cholula	McCormick
Sauce	Kikkoman	Kikkoman Corporation
Sauce	Thai kitchen	McCormick
Snack	Bimbo	Grupo Bimbo
Snack	Krusteaz	Continental Mills
Snack	Wetzel’s pretzel dogs	Center Oak Partners LLC
Snack	Bagel Bites	Kraft Heinz Company
Snack	Jell-o	Kraft Heinz Company
Snack	P3 portable protein snack	Kraft Heinz Company
Snack	Nature valley	General Mills
Snack	Toaster strudel	General Mills
Snack	Twinkies	Hostess Brands
Snack	Nutri grain	Kellogg’s
Snack	Pop-tarts	Kellogg’s
Snack	Jack link’s	Link Snacks, Inc
Snack	Clif bar	Mondelez International Inc
Snack	Wheat thins	Mondelez International Inc
Snack	Frigo cheese heads	Saputo Inc.
Snack	Pretzel crisps	Snack Factory LLC
Snack	Snyder’s pretzels	Snyder’s-Lance
Snack	Pistachios	The Wonderful Company
Snack	Kraft dinner	Kraft Heinz Company
Snack	Lunchables	Kraft Heinz Company
Snack	Blue almonds	Blue Diamond Growers
Snack	Goldfish	Campbell Soup Company
Snack	Kid cuisine	ConAgra Foods
Snack	RXBAR	Kellogg’s
Soda	Coca cola	The Coca-Cola Company
Soda	Sprite	The Coca-Cola Company
Soda	Canada dry	Keurig Dr Pepper
Soda	Crush	Keurig Dr Pepper
Soda	Dr. Pepper	Keurig Dr Pepper
Soda	Sunkist orange	Keurig Dr Pepper
Soda	Mountain dew	PepsiCo
Soda	Pepsi	PepsiCo
Sport drink	Powerade	The Coca-Cola Company
Sport drink	Gatorade	PepsiCo
Sweetened beverage	Smoothie king	
Sweetened beverage	Kool-aid	Kraft Heinz Company
Sweetened beverage	La croix	National Beverage Corporation
Sweetened beverage	Bubbly drops	PepsiCo
Sweetened beverage	Sparkling ice	Talking Rain Beverage Company
Sweetened beverage	Hawaiian sun	Hawaiian Sun Distributors LLC
Sweetened beverage	Nestea	Nestle
Sweetened juice	Minute maid	The Coca-Cola Company
Sweetened juice	Simply orange	The Coca-Cola Company
Sweetened juice	Simply smoothie	The Coca-Cola Company
Sweetened juice	Bai	Keurig Dr Pepper
Sweetened juice	Mott’s	Keurig Dr Pepper
Sweetened juice	SunRype	Sun-Rype Products Ltd
Sweetened juice	Slurpee	7-eleven
Sweetened juice	Juicy juice	Harvest Hill
Sweetened juice	Apple & Eve fruitables	Lassonde Industries Inc
Sweetened juice	Tropicana	PepsiCo
Sweetened juice	Welch’s	The National Grape Cooperative
Tea	Tazo	Unilever
Tomato sauce	Heinz ketchup	Kraft Heinz Company
Water	Vitamin water	The Coca-Cola Company
Water	Deer park water	Nestle
Yogurt	Chobani greek yogurt	Chobani
Yogurt	Gogurt	General Mills
Yogurt	Oikos	Danone
Yogurt	Oui yogurt	General Mills
Yogurt	Yoplait	General Mills
Yogurt	Siggi’s	Lactalis

## Appendix 2 OR, 95 % CI and *P*-values for the logistic regression model to predict whether food marketing instances appealed to children when the question is included as a predictor

**Table:**

Group	Variables	OR	95 % CI	*P* value
Question (‘Is this ad for kids your age?’ or ‘Is this ad for kids like you?’)	Question	1·00	1·00, 1·00	0·619
Food marketing instance attributes	Text*	1·02	1·01, 1·02	< 0·001
	Labels	1·00	0·99, 1·01	0·995
	Objects	1·00	1·00, 1·01	0·046
	Logos*	1·01	1·00, 1·01	0·002
Children’s characteristics	Age	0·98	0·88, 1·09	0·737
	Years living in Canada	0·99	0·93, 1·06	0·774
	Weight	1·00	0·97, 1·03	0·883
	Height	1·00	0·98, 1·02	0·962
	BMI z-score	0·98	0·88, 1·10	0·770
Children’s household characteristics	Household education	1·04	0·96, 1·12	0·334
	Household income	1·03	0·88, 1·20	0·757
	Perceived income adequacy	0·94	0·80, 1·11	0·459
Frequency of screen use	Weekly hours of television	1·09	0·98, 1·21	0·110
	Weekly hours of internet	0·91	0·77, 1·08	0·293
	Weekly hours of smartphone	1·01	0·73, 1·39	0·949
	Weekly hours of tablet/computer	0·89	0·79, 1·01	0·063
Dietary intake	Sodas per week	0·93	0·83, 1·05	0·258
	Chips per week	1·06	0·93, 1·21	0·372
	Candy/chocolate per week	1·01	0·93, 1·09	0·900
	Vegetables per week*	0·89	0·83, 0·96	0·002
	Fruits per week	0·98	0·89, 1·08	0·677
Sex	Male (female as reference)*	0·76	0·59, 0·99	0·038
Ethnicity	Asian (White as reference)*	1·39	1·09, 1·79	0·009
	Black (White as reference)	0·84	0·41, 1·70	0·624
	Latin American (White as reference)	0·84	0·38, 1·84	0·655
	South Asian (White as reference)	0·76	0·34, 1·67	0·489

*Means that the coefficient is statistically significant with an alpha set at 0·05.

## References

[1] Rideout VJ , Foehr UG & Roberts DF (2010) Generation M2: Media in the Lives of 8-to 18-Year-Olds. Henry J. Kaiser Family Foundation. Menlo Park, CA: ERIC.

[2] Montgomery KC , Chester J , Grier SA et al. (2012) The new threat of digital marketing. Pediatr Clin 59, 659–675. 10.1016/j.pcl.2012.03.022 22643172

[3] Kelly B , Vandevijvere S , Freeman B et al. (2015) New media but same old tricks: food marketing to children in the digital age. Curr Obes Rep 4, 37–45. 26627088 10.1007/s13679-014-0128-5

[4] Ali M , Blades M , Oates C et al. (2009) Young children’s ability to recognize advertisements in web page designs. Br J Dev Psychol 27, 71–83. 19972663 10.1348/026151008x388378

[5] Garriguet D (2009) Diet quality in Canada. Health Rep 20, 41. 19813438

[6] Proimos J & Klein JD (2012) Noncommunicable diseases in children and adolescents. Pediatric 130, 379–381. 10.1542/peds.2012-1475 22891233

[7] World Health Organization (2003) Diet, Nutrition, and the Prevention of Chronic Diseases: Report of a Joint WHO/FAO Expert Consultation, vol. 916. Geneva: World Health Organization. 12768890

[8] Taillie LS , Busey E , Stoltze FM et al. (2019) Governmental policies to reduce unhealthy food marketing to children. Nutr Rev 77, 787–816. 31329232 10.1093/nutrit/nuz021 PMC7528677

[9] Mulligan C , Potvin Kent M , Christoforou AK et al. (2020) Inventory of marketing techniques used in child-appealing food and beverage research: a rapid review. Int J Public Health 65, 1045–1055. 32840633 10.1007/s00038-020-01444-w

[10] Jenkin G , Madhvani N , Signal L et al. (2014) A systematic review of persuasive marketing techniques to promote food to children on television. Obes Rev 15, 281–293. 24433359 10.1111/obr.12141

[11] Mulligan C , Potvin Kent M , Vergeer L et al. (2021) Quantifying child-appeal: the development and mixed-methods validation of a methodology for evaluating child-appealing marketing on product packaging. Int J Environ Res Public Health 18, 4769. 33947116 10.3390/ijerph18094769 PMC8124606

[12] Elliott C & Truman E (2019) Measuring the power of food marketing to children: a review of recent literature. Curr Nutr Rep 8, 323–332. 31728913 10.1007/s13668-019-00292-2

[13] Mayhew AJ , Lock K , Kelishadi R et al. (2016) Nutrition labelling, marketing techniques, nutrition claims and health claims on chip and biscuit packages from sixteen countries. Public Health Nutr 19, 998–1007. 25818889 10.1017/S1368980015000658 PMC10271032

[14] Allemandi L , Castronuovo L , Tiscornia MV et al. (2018) Food advertising on Argentinean television: are ultra-processed foods in the lead? Public Health Nutr 21, 238–246. 28745262 10.1017/S1368980017001446 PMC10260822

[15] Harris JL , LoDolce M , Dembek C et al. (2015) Sweet promises: candy advertising to children and implications for industry self-regulation. Appetite 95, 585–592. 26232330 10.1016/j.appet.2015.07.028

[16] Elliott C (2009) Healthy food looks serious: how children interpret packaged food products. Can J Comm 34, 359–380.

[17] Chang R (2009) Mobile marketers target receptive Hispanic audience. Advert Age 80, 18.

[18] Young BM (1990) Television Advertising and Children. New York: Oxford University Press.

[19] Smith R , Kelly B , Yeatman H et al. (2019) Food marketing influences children’s attitudes, preferences and consumption: a systematic critical review. Nutrients 11, 875 31003489 10.3390/nu11040875 PMC6520952

[20] Boyland EJ , Kavanagh-Safran M & Halford JCG (2015) Exposure to ‘healthy’ fast food meal bundles in television advertisements promotes liking for fast food but not healthier choices in children. Br J Nutr 113, 1012–1018. 25716646 10.1017/S0007114515000082

[21] Esmaeilpour F , Heidarzadeh Hanzaee K , Mansourian Y et al. (2018) Children’s food choice: advertised food type, health knowledge and entertainment. J Food Prod Mark 24, 476–494.

[22] Pettigrew S , Tarabashkina L , Roberts M et al. (2013) The effects of television and Internet food advertising on parents and children. Public Health Nutr 16, 2205–2212. 23635396 10.1017/S1368980013001067 PMC10271572

[23] Baldwin HJ , Freeman B & Kelly B (2018) Like and share: associations between social media engagement and dietary choices in children. Public Health Nutr 21, 3210–3215. 30086811 10.1017/S1368980018001866 PMC10260990

[24] Tarabashkina L , Quester P & Crouch R (2016) Exploring the moderating effect of children’s nutritional knowledge on the relationship between product evaluations and food choice. Soc Sci Med 149, 145–152. 26717561 10.1016/j.socscimed.2015.11.046

[25] Bauer GR (2014) Incorporating intersectionality theory into population health research methodology: challenges and the potential to advance health equity. Soc Sci Med 110, 10–17. 24704889 10.1016/j.socscimed.2014.03.022

[26] Bzdok D , Altman N & Krzywinski M (2018) Statistics *v.* machine learning. Nat Methods 15, 233–234. 30100822 10.1038/nmeth.4642 PMC6082636

[27] Rajula HSR , Verlato G , Manchia M et al. (2020) Comparison of conventional statistical methods with machine learning in medicine: diagnosis, drug development, and treatment. Medicina (B Aires) 56, 455. 10.3390/medicina56090455 PMC7560135 32911665

[28] Valderrama C , Olstad D , Lee Y et al. (2022) What factors shape whether digital food marketing appeals to children? Curr Dev Nutr 6(Suppl. 1), 406–406. 10.1017/S1368980023000642 PMC10346072 37009657

[29] Statistics Canada (2016) Canadian Community Health Survey-Annual Component (CCHS). Ottawa, ON, Canada: Government of Canada.

[30] Hutchinson J , Williams T & Kirkpatrick SI (2021) Canada’s Food Guide What to Eat Screener Preliminary Report: Screener Development, Cognitive Testing, Face and Content Validity Testing, and Next Steps. A Report for Health Canada. Ottawa, ON: Health Canada.

[31] Hagenaars A & de Vos K (1988) The definition and measurement of poverty. J Hum Resour 23, 211–221.

[32] Litwin H & Sapir EV (2009) Perceived income adequacy among older adults in 12 countries: findings from the survey of health, ageing, and retirement in Europe. Gerontologist 49, 397–406. 19386829 10.1093/geront/gnp036 PMC2682171

[33] Common Sense (2021) The Common Sense Census: Media Use by Tweens and Teens. San Francisco, CA, USA: Common Sense.

[34] Byrne R , Terranova CO & Trost SG (2021) Measurement of screen time among young children aged 0–6 years: a systematic review. Obes Rev 22, e13260. 33960616 10.1111/obr.13260 PMC8365769

[35] Oracle Advertising (2011) MOAT. https://www.oracle.com/cx/advertising/measurement/ (accessed November 2020).

[36] Landis JR & Koch GG (1977) The measurement of observer agreement for categorical data. Biometrics 33, 159–174. 843571

[37] Bennett EM , Alpert R & Goldstein AC (1954) Communications through limited-response questioning. Public Opin Q 18, 303–308.

[38] World Health Organization (2007) Computation of Centiles and Z-Scores for Height-for-Age, Weight-for-Age and BMI-for-Age. Geneva: WHO.

[39] Google (2016) Vision API. https://cloud.google.com/vision/ (accessed January 2021).

[40] Seabold S & Perktold J (2010) Statsmodels: Econometric and Statistical Modeling with Python. In *Proceedings of the 9th Python in Science Conference* 57, pp. 10–25080. Austin, Texas, USA on June 28 and July 3, 2010. https://conference.scipy.org/proceedings/scipy2010/seabold.html (accessed December 2022).

[41] Pedregosa F , Varoquaux G , Gramfort A et al. (2011) Scikit-learn: Machine Learning in Python. J Mach Learn Res 12, 2825–2830.

[42] Strobl C , Hothorn T & Zeileis A (2009) Party on! R J 1, 14–17.

[43] Hosmer DW & Lemeshow S (2000) Applied Logistic Regression, 2nd ed. New York, NY, USA: Wiley.

[44] Hastie T , Tibshirani R , Friedman JH et al. (2009) The Elements of Statistical Learning: Data Mining, Inference, and Prediction, vol. 2. New York, NY, USA: Springer.

[45] Quinlan JR (1986) Induction of decision trees. Mach Learn 1, 81–106.

[46] Sutton CD (2005) Classification and regression trees, bagging, and boosting. Handb Stat 24, 303–329.

[47] Hothorn T , Hornik K & Zeileis A (2006) Unbiased recursive partitioning: a conditional inference framework. J Comput Graph Stat 15, 651–674.

[48] Artstein R & Poesio M (2008) Inter-coder agreement for computational linguistics. Comput Linguist 34, 555–596.

[49] Reidsma D & Carletta J (2008) Reliability measurement without limits. Comput Linguist 34, 319–326.

[50] Jamison E & Gurevych I (2015) Noise or Additional Information? Leveraging Crowdsource Annotation Item Agreement for Natural Language Tasks. In Proc*eedings of the* 2015 *Conference on Empirical Methods in Natural Language Processing (EMNLP)*. pp. 291–297. Lisbon, Portugal on September 17 and 21, 2015. Lisbon, Portugal: Association for Computational Linguistics. https://aclanthology.org/D15-1035/ (accessed December 2022).

[51] Leonardelli E , Menini S , Palmero Aprosio A et al. (2021) Agreeing to Disagree: Annotating Offensive Language Datasets with Annotators’ Disagreement. In *Proceedings of the 2021 Conference on Empirical Methods in Natural Language Processing.* pp. 10528–10539. Punta Cana, Dominican Republic on November 7 and November 11, 2021. Online and Punta Cana, Dominican Republic: Association for Computational Linguistics. https://aclanthology.org/2021.emnlp-main.822/ (accessed December 2022).

